# On the Present Condition and Treatment of Venereal Diseases in Paris

**Published:** 1851-02

**Authors:** William Acton

**Affiliations:** Late Surgeon to the Islington Dispensary, and Formerly Externe to the Venereal Hospitals of Paris


### SELECTIONS

FROM

### FOREIGN AND HOME MEDICAL JOURNALS.

---

*On the Present Condition and Treatment of Venereal Diseases in Paris.* From Notes taken during a recent visit to the French metropolis. By William Acton, Esq., late Surgeon to the Islington Dispensary, and formerly Externe to the Venereal Hospitals of Paris.—In preparing a second edition of my work on Venereal Diseases, I thought it advisable to pay a visit to the French capital, to observe for myself what improvements my late master, M. Ricord, had been making, during the last two years and a half, in the treatment of syphilis. Although the medical journals every now and then describe any new plan of treatment he may prescribe, still they give but an imperfect idea of the progress medical science is making; and the following notes may not prove uninteresting to the profession, founded, as they are, on the results of my own observation.

The civil authorities of Paris levy a duty on provisions entering the city. The tax goes towards defraying the expense of the hospitals, which are not supported by voluntary contributions, as in this country. The administration has founded large syphilitic institutions, both for men and women. The one I am about to describe is entirely given up to the treatment of men suffering under venereal diseases. The Hôpital du Midi contains nearly 400 beds, and there are at present three surgeons attached to it—M. Ricord, Vidal de Cassis, and Puche. The in-patients are treated gratuitously; the out-patients have only advice given—they are obliged to pay for the medicines, which are not dispensed at the institution. In most other French hospitals sisters of charity attend upon the sick; in this institution the duties are performed by men. The wards are very lofty and long, containing about twenty-five beds each. The committee require the patients to be seen daily; and it is the custom with French surgeons to go round the wards early in the morning, from eight to eleven. On Monday, in the wards of M. Ricord, every patient is seen; on Tuesday, only such as require any alteration in the treatment; on Wednesday, those that were admitted the previous day, when he himself, assisted by his house-surgeon, prescribes for all out-patients that apply. It struck me as singular, that since the revolution the students no longer take off their hats while in the wards, thus showing neither respect to the surgeon nor to the institution; yet that common mark of politeness of lifting the hat when entering a café, is kept up. Of course, in the wards of a Venereal Hospital, many are to be found who have taken, and will take, a part in a street row, barricade, or revolution; yet the greatest order reigns in the institution; any disturbance is met with immediate dismissal by the resident civil officer; and the patients well know that soldiers in the adjoining corps de garde would be instantly called in to expel any refractory patient, notwithstanding the words Liberté, Egalité, Fraterninté, written on the public buildings. The brotherly feeling, almost amounting to familiarity, observed by Ricord to his patients, is seldom abused; he will laugh at, or with a patient, ridicule another's imaginary fears, and the students will participate in his mirth, and the patient will smile; and all goes on smoothly. References are often made by all parties to the political events of the day, and censure is bestowed on the administration, the house-surgeon, dresser, or on the treatment followed in other hospitals; then comes a clinical lecture at the bedside of the patient, if the case is deserving of it; and the patient is cognisant of all that is said and done. Students are rather encouraged to put questions; and the wit and talent of the professor dreading no *contretemps*, everything is open to canvass; but impertinent observations are never indulged in, for were they employed, a laugh raised at the poor coxcomb's expense, would prevent a repetition of it—*mauvaise honte* and impertinence are equally absent. Let a patient, however, be refractory to treatment, or object to follow the prescribed routine, the light raillery gives place to just indignation, and he receives such a public admonition as serves for a warning to the inmates of the whole ward, who equally respect the kindness and the talent of the professor. The in-patients are recruited from the out-patients who are seen daily at the hospital, by one or other of the surgeons; they amount to enormous numbers, and the most severe cases are those admitted.

A surgeon attached to a large hospital, with a considerable private practice, is a very hard-worked man in Paris. Let me give you some idea of Ricord's way of spending his day. He leaves his home in the morning, between seven and eight; he first has to visit his Maison de Santé, which, I need not observe, is a private establishment for single persons who wish to have comforts and attendance they cannot procure when sick at their own homes; at eight he enters the wards of his hospital, and often remains there till eleven. His carriage is in waiting; and then he visits his patients in Paris until three o'clock; he now returns to a hasty breakfast, and is occupied at home until seven, eight, or ten o'clock in the evening; and he assured me that he was rarely able to dine out, in consequence of his patients occnpying his time to so late an hour. He has now to answer his correspondents, and prepare matter for the press, and here for the first time he can obtain assistance. Now, although Ricord is nearly fifty, and has led this sort of life, to my knowledge, for twelve years, he is as young-looking as ever; his brown hair remains unchanged; he has no careworn appearance, but is as cheerful as ever; the same sparkling wit as formerly; and never has a surgeon been more adored by his patients, who feel convinced that he does nothing all day but consider their own particular complaints.

A French surgeon's consultation room is well adapted to the purpose: placed in the centre of several others, with doors opening into each, patients are admitted and divided into little parties. *Egalité* is not strictly observed here, particularly if the servant be feed, a practice as common in Paris as it is in London consultation rooms. The rich man need not wait so long as he who has only just enough to fee the surgeon. Late events in Paris have caused a great diminution in the practice of the French surgeon; he is called on to give a great deal of gratuitous advice. M. Ricord tells me he has always made it a rule to prescribe for patients who apply; and he never demands a fee, but on his table are seen sums varying from ten francs to Napoleons and sovereigns, which patients place there on leaving.

Asking pardon for this digression, I must return to the more proper subject of this paper. The 2d of April, 1850, the day I visited M. Ricord's wards, was Easter Monday, and the French, like the English, make it a holiday; there were a few beds vacant, but the following is the *resumé* of the cases.

| | |
|---|---|
| Indurated chancre - - - - - | 33 |
| Secondary symptoms - - - - | 21 |
| Bubo - - - - - - - - - | 8 |
| Vesicle catarrh - - - - - - | 4 |
| Phagedænic chancre - - - - | 2 |
| Epididymitis - - - - - - | 7 |
| Urinary fistula - - - - - | 1 |
| Vegetations - - - - - - | 2 |
| Tertiary symptoms - - - - | 8 |
| Iritis - - - - - - - - | 1 |
| Simple chancre, non-indurated - - | 11 |
| Gonorrhœa præputialis - - - | 3 |
| Scrofulous affection of the testis - - | 1 |
| Blennorrhagia - - - - - - | 3 |
| Stricture - - - - - - - | 1 |
| Chancre of the anus - - - - | 3 |
| Gonorrhœal rheumatism - - - | 1 |
| Albuminuria - - - - - - | 1 |
| Hæmorrhoids - - - - - - | 1 |
| | 112 |

The first thing which strikes the most casual observer, on loooking over the cases, is the large proportions of indurated chancres—thirty-three cases in the wards at one time. Here the student may study induration in all its forms. The frequency of this symptom in the wards depends upon M. Ricord admitting by preference, as patients, those who suffer under this form of chancre. Like all writers on syphilis, he lays the greatest stress on this symptom; but differing from all his predecessors, he does not consider all hardness attending a chancre to be induration. To diagnose this true symytom of syphilis, he looks for, and points out to the pupils, the enlargement of the glands in the groins, occurring a few days after the induration on the penis. These buboes rarely suppurate; when matter forms it is the result of inflammation superadded to the disease, and is an accident, whereas the enlargement in the groin is a constant attendant upon indurated chancre. When this symptom is not to be detected, he often keeps the patient in the wards, to show that the false ulceration will subside of itself, and secondary symptoms not occur.

I said above that you may study, in these thirty-three cases of indurated chancre, the disease in all its varied consequences. In by far the majority of cases, secondary symptoms had already appeared; and on reference to the table, twenty-one cases are noted down in which secondary symptoms were the most prominent feature; here indurated chancre had previously existed. In these cases of constitutional syphilis, M. Ricord dwells particularly on a character not noticed by previous writers—namely, enlargement of the glands at the back of the neck, a very constant occurrence in secondary symptoms, and one that comes on early—say five weeks—after general contamination of the system. The treatment of all these cases consists in ordering the mineral, in the form of proto-ioduret of mercury, a preparation he almost exclusively gives, but one which in England I have been unable to prescribe, as the same pills which patients bring from Paris act too violently on the bowels in this country, inducing colic and diarrhea,—consequences rarely seen in Paris, and which we must attribute to climate and difference in diet in the two capitals. I need not here dwell on the long period mercury, he thinks, must be given in cases of indurated chancre or secondary symptoms, and continued even after their disappearance; three or six months' persistence will scarcely be long enough to eradicate the syphilitic diathesis, and prevent relapses when the constitution has been once infected. All his further experience has only convinced the professor of the Hôpital du Midi of the correctness of his ideas long since given to the public, but he now further assists their action by prescribing more frequently.

*Fumigations.*—This mode of giving mercury is so rarely followed in London, at the present day, that perhaps few of my readers are aware of the means employed.

The patient is placed naked in a box, his head being the only part exposed to the air. This box is heated by a furnace below, on which the bisulphuret of mercury is placed, in proportions of three drachms. The heat soon volatilizes the mineral, and the fumes come in contact with the skin. The patient is allowed to remain twenty minutes in a box, and a tumblerful of water is given him. In a few minutes perspiration comes on, and the patient is then taken out and well rubbed down; and the fumigation may be repeated twice a week for a month or six weeks, according to circumstances. In old chronic affections which do not yield to mercury, given internally, the most marvellous effects are often produced, and I have seen cases (resisting all other treatment) get rapidly well under this plan; and I would recommend a trial to my professional brethren in the numerous instances that come before them of obstinate complaints. I am, however, by no means convinced of its general application, or that it is likely to supersede the mineral, given internally. It has been said that patients may employ fumigations, and yet pursue their ordinary occupations. One case that has lately come under my notice induces me to recommend that patients, after employing the cinnabar, should not expose themselves to the open air. I fear, likewise, that in country practice the use of it must be circumscribed. A fixed apparatus will be necessary, as the volatilization of the mercury is difficult, in consequence of the great quantity of heat required. The ordinary spirit-lamp appears insufficient. I lately placed a patient in a vapor-bath, and after weighing three drachms of the cinnabar, placed it on the plate, over a strong spirit-lamp. In twenty minutes the substance had only lost one scruple in weight, independently of waste, thus showing that this is a very imperfect way of volatilizing the mercury, the vapor of which is so heavy that it will not rise more than one foot, unless carried up by a strong current of air. These peculiarities explain why fumigations have fallen into disuse, as the proper means of application are not always at hand.

*Tertiary Symptoms* may be seen in the wards in all their variety; and I noticed one which will form the subject of a separate communication; viz: of amaurosis, depending upon disease of the sphenoid bone. Suffice it, at present, to say that iodide of potash is our sheet-anchor, given *in large doses*. It is a very general belief in London, among our leading surgeons, that all the good that iodide of potash can do, will accrue if three or five grains be given, three times a day; more than that, I am daily told, will do harm, or if more be given with impunity, the article is spurious. I do not wish to underrate the good effects of the salt often resulting from small doses, but I am as equally convinced that large doses may be given with impunity. I lately had a gentleman from Scotland under my care, who took the remedy in such quantities that he purchased it by the half pound, and yet it was a genuine article; but I will go further than this, and assert, from a pretty large experience of its effects, that small doses will not do any or the slightest good in many instances. I have now under my care a patient with affection of the nose, and destruction of the ossi nasi, who had been taking these small doses of iodide of potash for many months, under a late lamented surgeon, and the disease remained pretty nearly in *statu quo*. She came under my care, and the affection is now on the point of complete recovery under increased doses.

I met Mr. Wallace in consulation a short time since, in reference to a case of tertiary symptoms of the nose and brow, which iodine had relieved, but not cured, although taken in these small doses by direction of another surgeon for nine months; here the salt had been obliged to be left off, because iodic intoxication was said to have been produced, together with symptoms of affection of the brain. Now in these instances, time, rather than the dose, was in fault, and the surgeon who entertains these ideas scruples not to prescribe iodide of potash for years, rather than give it in larger doses.

I saw, with Mr. Vickers, a gentleman who has now entirely recovered, to whom we gave large doses of iodide of potash with bitters, yet small doses had failed in curing him, although given for long periods under the advice of other practitioners. I mention these cases because prejudices exist against the employment of the remedy, whereas, in France, Ricord gives it in anything but homœopathic doses, and with the most signal success.

*Phagedænic Chancre.*—At the time I visited the wards, only two cases existed of this disease, but generally it is much more frequently met with. Under this head may be included all those anomalous sores met with in London, or elsewhere, which are classed as irritable chancres, occurring in bad constitutions, indisposed to heal, spreading, instead of healing kindly, and causing the greatest annoyance to both surgeon and patient. In consultation practice in London, these are the cases I am most frequently consulted about to sanction the use of mercury. All sorts of washes have been used, caustic has been applied, but without avail, and the case is getting worse. In all such instances, iron is the remedy, and its effects are perfectly magical. I need not say that mercury aggravates such cases. Of all preparations, the tartrate of iron is best given in the following manner:—potassio tartrate of iron, 1 ounce; water, 6 ounces. Mix. Two tablespoonsful three times a day.

M. Ricord largely employs it, and prescribes it now, even in the treatment of simple cases of chancre; he uses it, likewise, as a local application, as well as an internal remedy.

*Chronic Catarrh of the Bladder.*—Ricord now uses caustic injections into the viscus itself. Having emptied the organ of urine, he passes a gum-elastic catheter, and then, with a glass syringe, throws the injection into the bladder, and repeats it, according to circumstances, every three or four or six days. There is some pain and irritation set up; the urine becomes a little bloody, but the ropy secretions soon ceases. The different preparations of turpentine are given, and I saw four cases in their different stages. He has not met with any ill consequences of the treatment, although the injections used are composed of nitrate of silver, 2 drachms; distilled water, 4 ounces. Mix for an injection.

*Strictures.*—On the day I took notes of the cases, only three instances of stricture of the urethra presented themselves in M. Ricord's wards, and one of urinary fistula. The whole of these were serious examples of their respective diseases, and formed the subject of a clinical lecture. I am happy to say that the eminent Professor of the Hôpital du Midi corroborated many of the opinions I expressed during the last winter, on the subject of treatment. In the ordinary cases, he employs simple dilatation by means of conical gum-elastic bougies, with a little olive-shaped point, such as I now produce; when retraction takes place, the case is treated by incision with instruments passed down the urethra, and he has lately introduced several modifications in the shape of the instruments with which the stricture is to be incised. Nitrate of silver is rarely employed in stricture; and on the subject of potassa fusa, M. Ricord agrees with modern surgeons, that in small quantities the kali becomes saponified, and is insufficient; that in larger masses, when pressed against the obstruction, it is attended with the most serious consequences and is not a remedy at all adapted to overcome the impassable strictures which every now and then come before the surgeon.

I mentioned to M. Ricord the fatal results which had in London attended the operation of incising the perineum as recommended by Professor Syme. He thought that one or two unsuccessful cases should not deter us from prosecuting the plan, provided all others had failed. He acknowledged that very puzzling cases every now and then came before us, and he cited an instance in his own practice, where death followed from incising the stricture by instruments introduced through the urethra, the fatal result (just as in the London cases) following from typhoid symptoms.

But the numerous late instances of repeated deaths pointed out the caution that should be exercised, and he was by no means convinced that the stricture would not return after a certain period. Still, (added he, in his peculiar laughing way, which often winds up a learned disquision on a plan of treatment,) I should much rather find my patient complaining of the return of the stricture, than the surgeon regretting the non-return of his patient, as you state is the case in London.

I regret being obliged to omit many important observations which I made during my stay, as well as other remarks on the out-patients, but I hope I have said enough to show how much society and the medical profession are indebted to M. Ricord for the manner in which he has successfully carried out the treatment of venereal diseases, and following in the footsteps of the immortal John Hunter, has recalled the attention of the profession to a class of diseases that of late years had become the especial domain of quacks.—*London Lancet*, December, 1850.

